# Is Commercial Air Travel Safe in Thoracic Aortic Disease? A Physiological and Clinical Perspective

**DOI:** 10.3390/jcdd13070319

**Published:** 2026-07-09

**Authors:** Nimrat Grewal, John A. Elefteriades, Matthew M. Cooper

**Affiliations:** 1 Department of Cardiothoracic Surgery, Amsterdam University Medical Center Location AMC, Meibergdreef 9, 1105 Amsterdam, The Netherlands; 2 Aortic Institute at Yale New Haven Hospital, Yale University School of Medicine, 333 Cedar St, New Haven, CT 06510, USA; john.elefteriades@yale.edu; 3 Peak Aerospace Medicine & Cardiovascular Surgery, Lake Elmo, MN 55042, USA; mmcoopermd@gmail.com

**Keywords:** thoracic aortic disease, aortic aneurysm, aortic dissection, air travel, blood pressure, risk assessment

## Abstract

Patients with thoracic aortic disease (TAD) frequently seek advice on the safety of commercial air travel. Despite the clinical relevance of this question, robust evidence is virtually absent, and current recommendations rely largely on expert opinion. This Viewpoint discusses the physiological effects of flights, reviews the limited available evidence, and proposes a pragmatic, risk-based approach for clinicians counselling patients with TAD.

## 1. Introduction

Thoracic aortic disease encompasses a heterogeneous spectrum, including thoracic aortic aneurysm, bicuspid aortic valve-associated aortopathy, heritable thoracic aortic disease, and survivors of acute aortic syndromes. Advances in surveillance, genetics, and surgical care have led to a growing population of patients living with chronic aortic pathology.

In daily practice, one question recurs with striking frequency: “Is it safe for me to fly?” Unlike exercise, pregnancy, or blood pressure management, air travel is rarely addressed in major cardiovascular guidelines [1]. As a result, advice varies widely, often reflecting uncertainty rather than evidence. This lack of clarity risks both unnecessary restriction and inappropriate reassurance.

In contrast to many other aspects of thoracic aortic care, this question places the clinician in a particularly uncomfortable position. Patients typically expect a binary answer, yet the physician is forced to operate in an evidence vacuum. The medicolegal implications of advice that cannot be anchored in data further contribute to conservative counselling. As a result, practice patterns vary widely, even among experienced aortic specialists. Making this implicit reasoning explicit is therefore essential to more consistent and transparent care.

Although thoracic aortic disease is not addressed specifically, several organisations, including the American Heart Association, the International Air Transport Association (IATA), the Federal Aviation Administration, the Civil Aviation Authority, and the Aerospace Medical Association, have issued fitness-to-fly guidance for related cardiovascular and vascular conditions, including abdominal aortic aneurysm [2,3,4,5,6]. It is also useful to place the concern in an epidemiological context: in-flight medical emergencies are uncommon and serious cardiovascular events rarer still, with historical estimates of approximately 0.31 in-flight deaths per million passengers carried [7] and 1.52 medical diversions per billion revenue passenger-miles flown [8]. Aortic emergencies specifically are exceedingly rare among such events, which underscores both the scarcity of direct data and the need for proportionate, individualised advice.

## 2. Physiological Effects of Commercial Flight

Modern commercial aircraft are pressurized to a cabin altitude of approximately 6000–8000 ft [9]. This results in reduced inspired oxygen tension and mild hypobaric hypoxia. In healthy individuals, this typically leads to modest reductions in arterial oxygen saturation and compensatory increases in heart rate and cardiac output, without clinically relevant increases in systemic arterial pressure [10]. The classic aviation medicine literature consistently emphasizes that cabin pressurization primarily affects oxygenation rather than intravascular pressure dynamics.

The clinical implications of this mild hypobaric hypoxaemia nonetheless warrant consideration. The reduction in inspired oxygen tension elicits a compensatory sympathetic response, with increases in heart rate, cardiac output, and myocardial oxygen demand, and can produce modest changes in vascular tone and systemic blood pressure. In healthy individuals, these adaptations are well tolerated. In patients with advanced thoracic aortic disease or significant cardiovascular comorbidity, however, the same sympathetically mediated haemodynamic changes could theoretically augment aortic wall stress, particularly where blood pressure control is already marginal. This provides a further rationale for optimising haemodynamic and oxygenation status before travel in higher-risk phenotypes, and, where indicated, for pre-flight assessment of the need for supplemental oxygen in those with coexisting cardiorespiratory disease [5,6].

From a mechanical standpoint, aortic wall stress is determined predominantly by transmural pressure and vessel geometry. The modest changes in ambient pressure encountered during commercial flight translate into minimal alterations in intravascular pressure relative to the fluctuations routinely observed during activities such as climbing stairs, emotional arousal, or isometric exertion. Furthermore, the thoracic aorta is continuously exposed to pulsatile stress far exceeding any static pressure changes induced by cabin altitude. It is therefore difficult to construct a plausible biomechanical scenario in which cabin pressurization, in isolation, would constitute a dominant trigger for aortic failure.

## 3. Triggers of Acute Aortic Events and the Relevance of Travel

Observational studies and registry data consistently identify uncontrolled hypertension, sudden physical exertion, and intense emotional stress as common antecedents of acute aortic syndromes [11]. These triggers act predominantly through abrupt elevations in systolic blood pressure and shear stress.

While flight itself does not appear to induce such extremes, the broader context of travel may expose patients to several recognised stressors: carrying heavy luggage, time pressure, sleep deprivation, dehydration, anxiety, and disruption of medication schedules. It is therefore plausible that the process of travel, rather than the cabin environment, constitutes the more relevant risk context for susceptible patients.

## 4. What Does the Clinical Literature Show?

To date, there are no prospective studies or large observational cohorts evaluating thoracic aortic outcomes in relation to commercial air travel. The available literature is limited to isolated case reports describing temporal associations between flying and acute aortic dissection [12,13]. These reports cannot establish causality and are subject to substantial publication bias.

Two caveats regarding this literature deserve emphasis. The single dissection report frequently cited in this context [12] concerns an acute type A dissection in a fighter pilot precipitated by an acute high-G “spiral-down” manoeuvre; this represents acceleration-induced injury in a military aircraft rather than a pressurisation-related event in a commercial cabin, and its relevance to commercial air travel is therefore limited. For abdominal aortic aneurysm, the available report [13] does not present primary data establishing safety: no study has demonstrated an increased rupture risk attributable to air travel, but the question remains formally unproven owing to a lack of data rather than to evidence of absence. As a Viewpoint rather than a systematic review, this article draws on a focused, non-systematic search of PubMed and Google Scholar to May 2025 (combining the terms “air travel”, “flight”, “cabin pressure”, “thoracic aortic aneurysm”, and “aortic dissection”), supplemented by manual review of reference lists and relevant society guidance; we acknowledge that some individual reports may not have been captured.

## 5. The Fallacy of Anecdote

Case reports of dissection occurring during or shortly after air travel are often compelling, yet they illustrate a classic post hoc fallacy. Given the background incidence of acute aortic syndromes and the large number of individuals with known or undiagnosed aortic disease who travel each day, temporal coincidence is inevitable. Without denominator data, such reports risk reinforcing fear rather than informing risk.

In contrast, no study of abdominal aortic aneurysm populations has demonstrated an increased rupture risk attributable to air travel; this reflects an absence of evidence rather than evidence of absence, as the available data are too sparse to exclude such a risk [6]. With that important caveat, the lack of any demonstrated signal is at least consistent with the concept that hypobaric cabin conditions alone are unlikely to represent a dominant trigger, while anatomical and pathophysiological differences preclude direct extrapolation to the thoracic aorta.

## 6. Clinical Interpretation

On the basis of current knowledge, commercial air travel should not be regarded as an independent precipitant of thoracic aortic complications. The more plausible mechanism of risk relates to peri-travel blood pressure variability and haemodynamic stress rather than to the flight environment itself [14].

This peri-travel blood pressure variability is likely multifactorial. Plausible contributors include sympathetic activation driven by anticipatory anxiety and emotional stress, sleep disruption and circadian misalignment, missed or mistimed antihypertensive medication, dehydration, and the physical exertion of carrying luggage and navigating busy terminals. Individually and cumulatively, these factors can provoke transient surges in systolic blood pressure and sustained haemodynamic instability. Because blood pressure variability is itself an established predictor of cardiovascular events independent of mean pressure [14], it is these peri-travel fluctuations, rather than the cabin environment per se, that represent the most plausible and, importantly, the most modifiable source of risk during air travel.

It is, however, unlikely that risk is uniform across all phenotypes. Patients with heritable aortopathies, marked aortic fragility, or pronounced blood pressure lability may be more susceptible to haemodynamic stressors. In these subgroups, even modest triggers could, in theory, contribute to adverse events. The absence of evidence in these populations should prompt caution, but not automatic prohibition, emphasizing the importance of individualized assessment.

**A pragmatic risk-based approach.**

**Patients generally suitable for air travel:**

- Stable thoracic aortic aneurysm without rapid growth (rapid growth defined as an increase of ≥5 mm over 6 months, or ≥3 mm per year);
- Well-controlled arterial hypertension;
- Chronic, stable patients after surgical or endovascular repair;
- Absence of recent aortic symptoms or acute aortic syndromes.

**Patients in whom caution or temporary deferral is reasonable:**

- Recent acute aortic syndrome (dissection, intramural haematoma, or penetrating ulcer), typically within the preceding 1–3 months.
- Rapid aneurysm expansion.
- Aortic diameters approaching or exceeding accepted intervention thresholds.
- Poorly controlled blood pressure

The early postoperative phase, particularly after recent open surgery, where residual respiratory compromise, pneumothorax, or anaemia may further reduce physiological reserve and warrant additional caution. Within this framework, hypertension should be regarded as a high-risk feature only when poorly controlled; well-treated, stable hypertension does not by itself contraindicate flying. Two further considerations merit emphasis. First, flight duration may modify risk: short-haul flights (≤3 h) entail briefer exposure to hypobaric conditions and immobility than long-haul flights (>3 h), and for higher-risk patients, a graded approach, favouring shorter sectors and deferring prolonged long-haul travel until the patient is stabilized, is reasonable. Second, patients with thoracic aortic disease frequently have concomitant cardiovascular and respiratory disease; coexisting coronary artery disease, heart failure, arrhythmia, and chronic lung disease should be identified and optimised as part of any pre-travel evaluation, in line with existing cardiovascular and respiratory fitness-to-fly guidance [4,5].

## 7. Practical Counselling

When air travel is considered acceptable, clinicians should emphasise strict blood pressure control and medication adherence, avoidance of heavy lifting and straining, particularly activities associated with Valsalva-type manoeuvres, as well as strenuous exertion during travel, adequate hydration, minimisation of psychological stress and time pressure, and the importance of carrying a concise medical summary including diagnosis and recent imaging.

These principles can be operationalised as a practical pre-travel checklist: (i) confirm stable, well-controlled blood pressure and full medication adherence in the days before travel; (ii) avoid heavy lifting, straining, and Valsalva-type manoeuvres, and request luggage assistance; (iii) arrange airport wheelchair or mobility assistance for frail or recently treated patients; (iv) avoid excessive alcohol consumption and maintain adequate hydration to limit haemodynamic swings; (v) carry an adequate supply of all medications in hand luggage, with a buffer for delays; (vi) obtain appropriate travel insurance with explicit cover for the pre-existing aortic condition; and (vii) carry a concise medical summary (diagnosis, most recent imaging, treatment, and current medication) and wear medical alert identification. For higher-risk patients, the need for supplemental in-flight oxygen or an airline medical clearance form should be considered in advance.

## 8. Implications for Guidelines and Patient Communication

The persistent uncertainty surrounding air travel in thoracic aortic disease highlights a broader issue in guideline development. Topics that lack randomized or large observational data are often omitted, yet these are precisely the questions that dominate patient consultations. Explicitly acknowledging uncertainty, while offering physiology-based guidance, may be preferable to silence. For patients, transparent communication that distinguishes between theoretical risk and proven harm is essential to support informed, shared decision-making.

Consistent with IATA guidance, patients with thoracic aortic aneurysm or thoracic aortic disease should ideally be assessed by a medical professional before flying to determine fitness for travel [3]. Such a specialist review should equip the patient with a concise summary of their diagnosis and treatment, recent investigations, and current medication, and patients should be advised to carry this documentation and to wear medical alert identification. Pre-travel review is especially important for individuals who have been lost to follow-up, who lack a recent assessment of symptoms or up-to-date imaging surveillance, or who are awaiting surgery or re-intervention, as these are the groups in whom unrecognised disease progression is most likely.

## 9. Knowledge Gaps and Future Directions

The absence of systematic data represents a major limitation. Prospective registries capturing recent travel exposure prior to acute aortic events, combined with detailed phenotyping and wearable-derived haemodynamic data, would substantially advance risk stratification. Meaningful progress will require international collaboration between aortic specialists, hypertension experts, and aviation medicine physicians.

## 10. Conclusions

There is no convincing evidence that commercial air travel increases the risk of thoracic aortic complications per se. Until robust data become available, counselling should be grounded in physiological reasoning, individualized risk assessment, and mitigation of peri-travel stressors rather than blanket restrictions on flying.

## Data Availability

No new data were created or analyzed in this study.
